# Supplementary figures and images for: Naïve Primary Mouse CD8+ T Cells Retain In Vivo Immune Responsiveness After Electroporation-Based CRISPR/Cas9 Genetic Engineering
Source: Front Immunol. 2022 Jun 30;13:777113. doi: 10.3389/fimmu.2022.777113 (PMC9280190; doi:10.3389/fimmu.2022.777113)

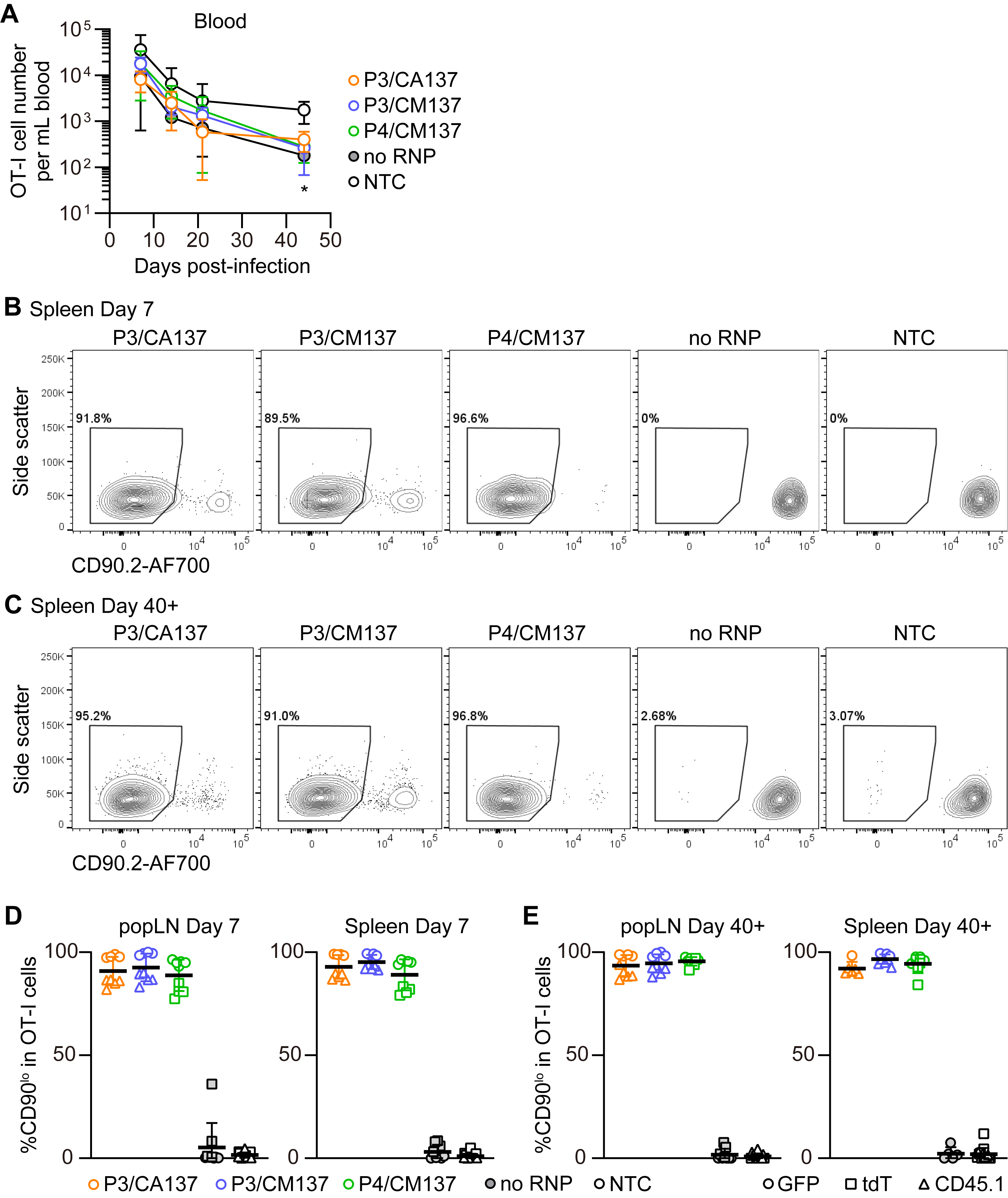

Supplement: Supplementary Figure 1 — Kinetics of the number and CD90 expression of in vitro-activated OT-I cells after adoptive transfer into HSV-OVA-infected hosts. Nucleofection of OT-I cells and viral infection were performed as in. (A) Kinetics of OT-I cell number per 1 mL blood. Statistical significance of differences between each of nucleofected group and NTC was analyzed by ordinary two-way ANOVA with Dunnett’s multiple comparison. *p < 0.0001. (B–E) Frequency of CD90lo cells among OT-I cells in popLN and spleen on days 7 (B, D) and > 40 (C, E). Congenic marker assignment was swapped in each experiment. Data are pooled from two independent experiments with n = 6–9 or n = 12–15 for nucleofected cells or non-nucleofected control, respectively. [file Image_1.tif]

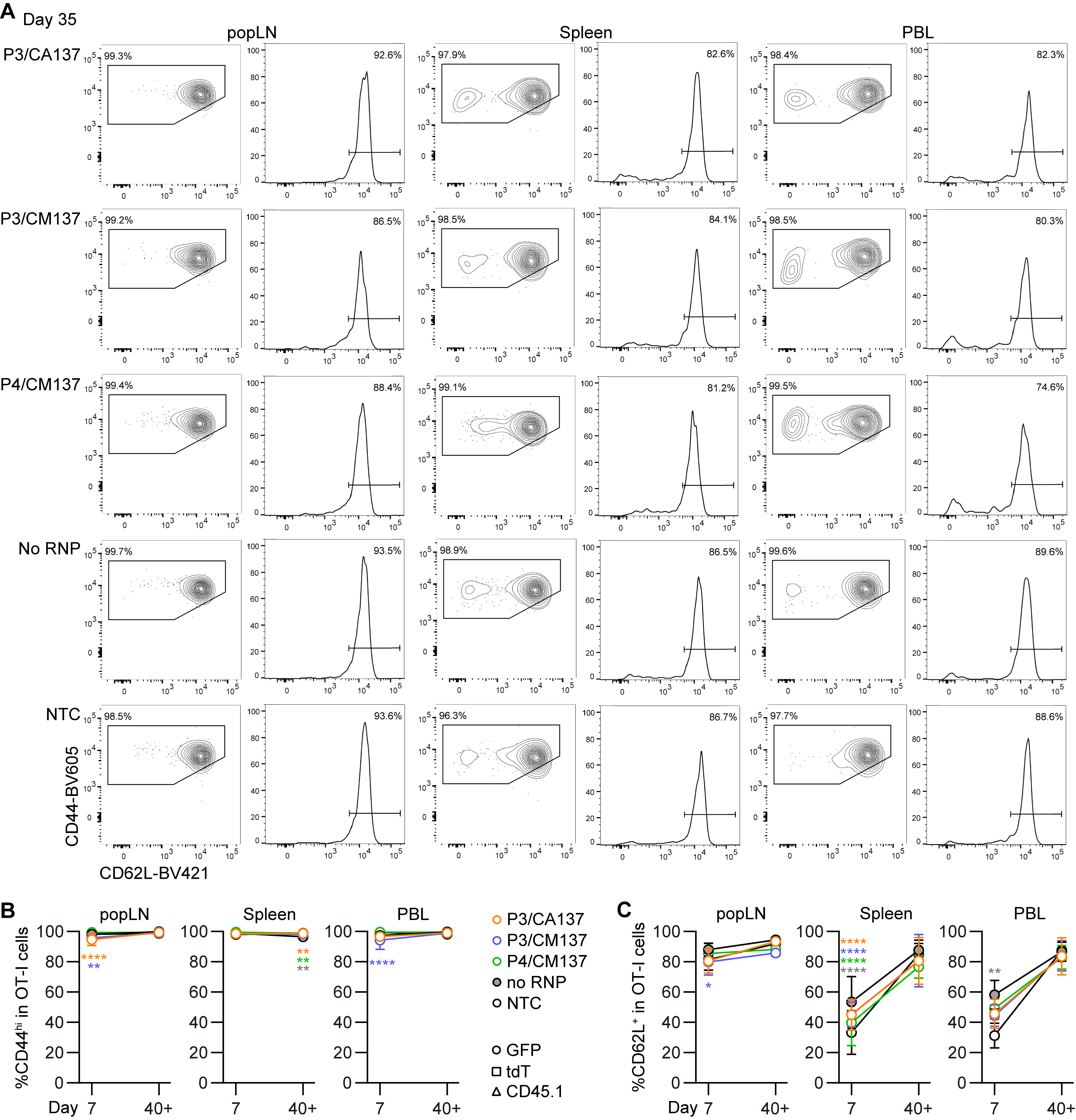

Supplement: Supplementary Figure 2 — Expression of CD44 and CD62L on in vitro-activated OT-I cells after adoptive transfer into HSV-OVA-infected hosts. (A) Representative plots showing the expression of CD44 and CD62L on OT-I cells in popLN, spleen and PBL. Plots show concatenated data of one of two experiments shown in. (B, C) Graph summary of the frequency of CD44hi (B) and CD62Lhi (C) OT-I cells. Graphs show pooled data from two independent experiments with n = 5–10. *p < 0.05, **p < 0.01, ***p < 0.001, ****p < 0.0001 as compared to NTC by ordinary two-way ANOVA with Dunnett’s multiple comparison. [file Image_2.tif]

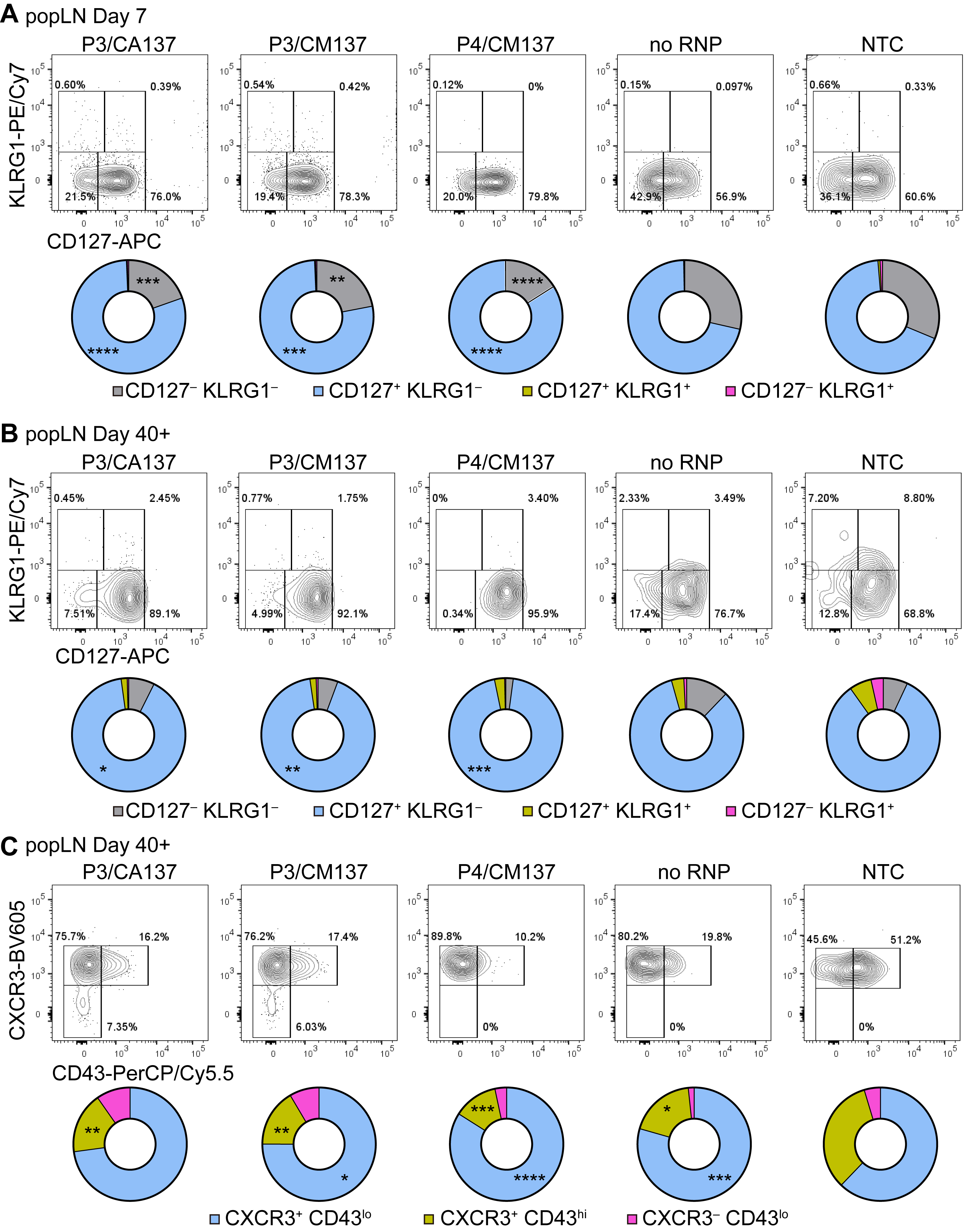

Supplement: Supplementary Figure 3 — Phenotype of in vitro-activated OT-I cells in popLN after adoptive transfer into HSV-OVA-infected hosts. Nucleofection of OT-I cells and viral infection were performed as in . (A, B) Expression of CD127 and KLRG1 on OT-I cells in popLN on days 7 (A) and > 40 (B). Pie charts show the mean frequencies of four populations identified by these two markers. (C) Expression of activation-associated glycoform of CD43 and CXCR3 on OT-I cells in popLN > 40 days after infection. Pie charts show the mean frequencies of three populations identified by these two markers. Graphs show pooled data from two independent experiments with n = 9 or 15 for nucleofected cells or non-nucleofected control, respectively. Flow cytometric plots are gated on viable OT-I cells identified by the expression of congenic markers and show concatenated data from one of two experiments with n = 5 per group. Congenic marker assignment was swapped in each experiment. *p < 0.05, **p < 0.01, ***p < 0.001, ****p < 0.0001 as compared to NTC by ordinary two-way ANOVA with Dunnett’s multiple comparison. [file Image_3.tif]

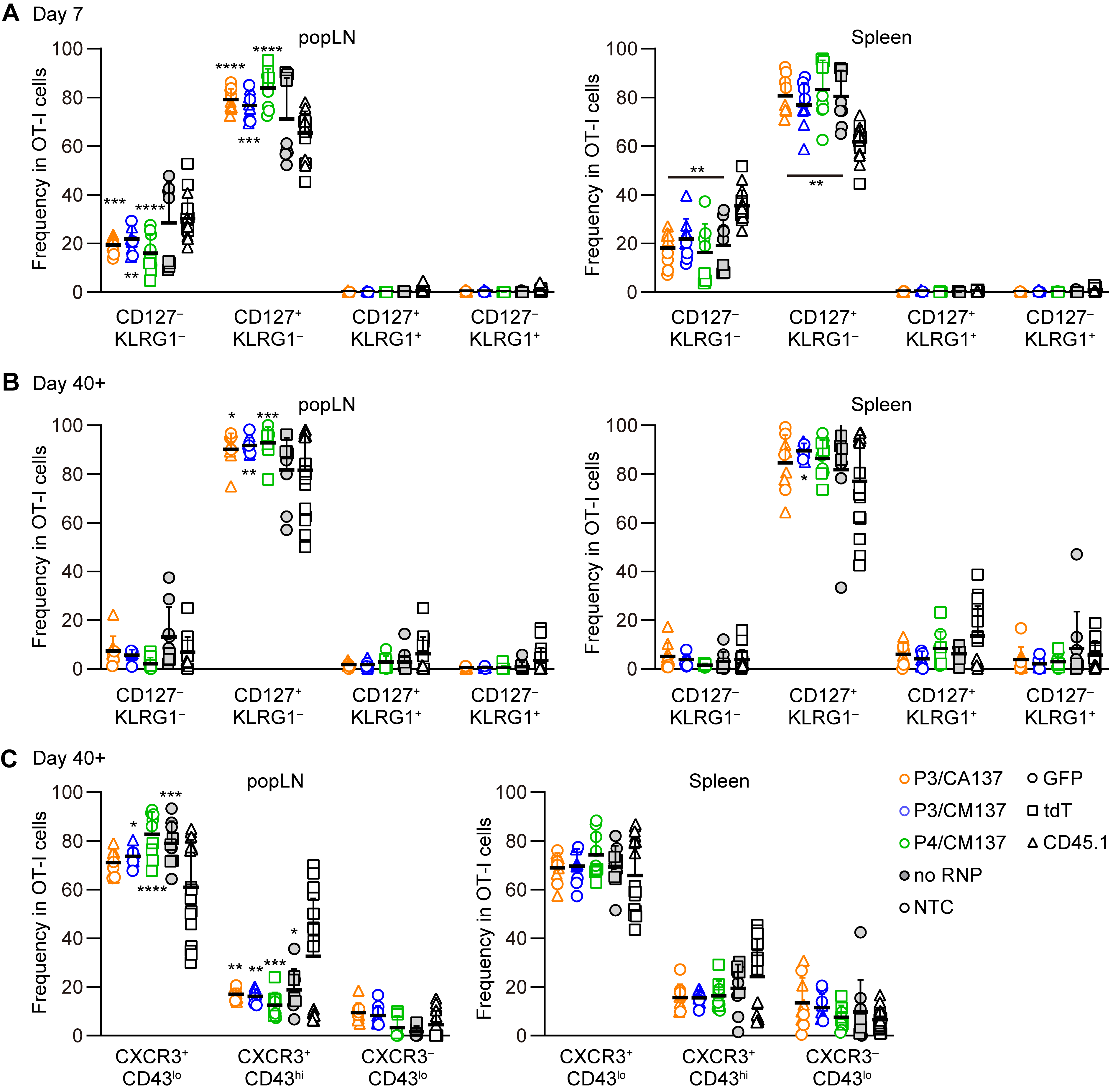

Supplement: Supplementary Figure 4 — Scatter plots of the data shown as pie charts in . (A, B) Frequency of four subsets defined by the expression of CD127 and KLRG1 on day 7 (A) and >40 (B). (C) Frequency of three subsets defined by the expression of activation-associated glycoform of CD43 and CXCR3. *p < 0.05, **p < 0.01, ***p < 0.001, ****p < 0.0001 as compared to NTC by ordinary two-way ANOVA with Dunnett’s multiple comparison. [file Image_4.tif]

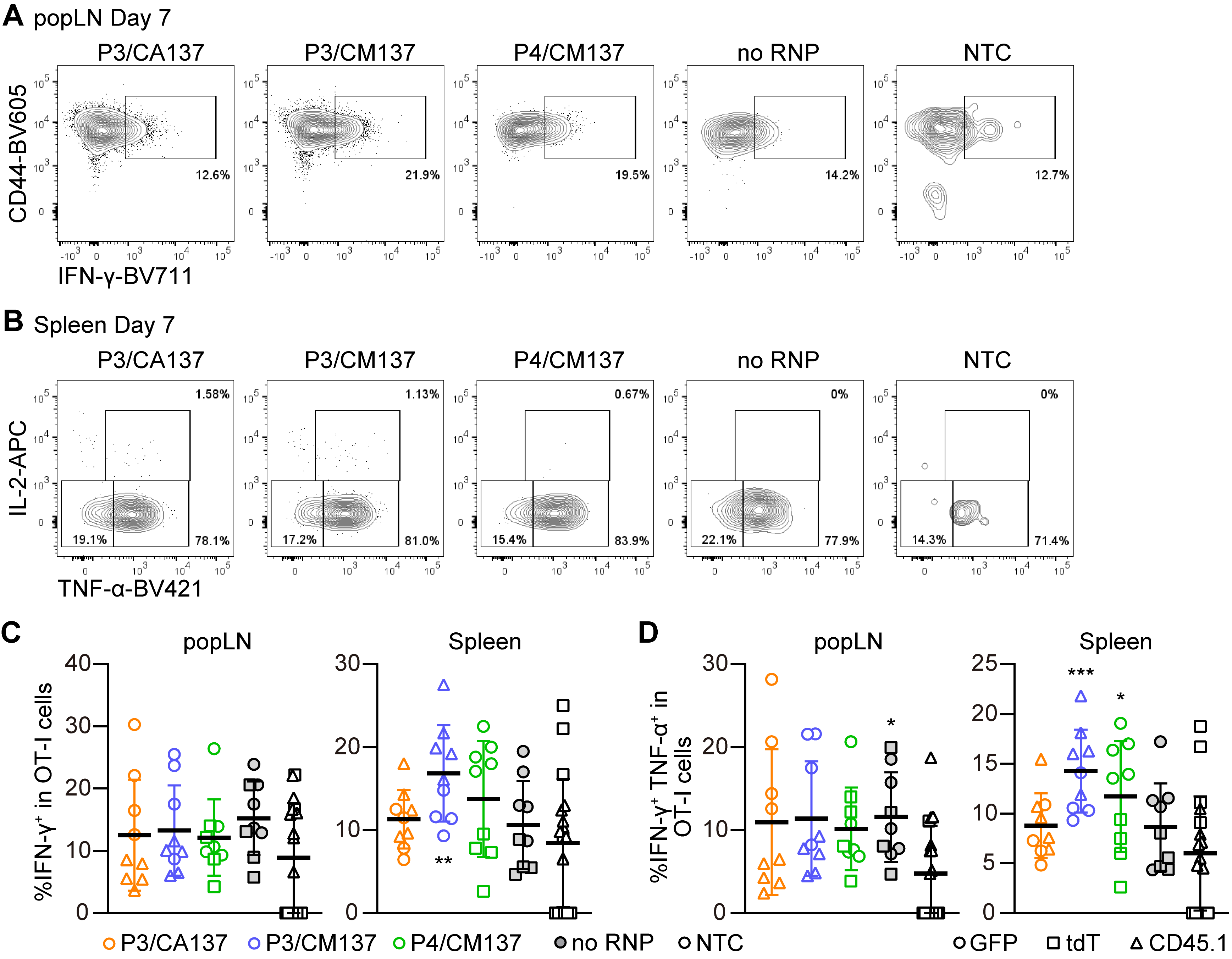

Supplement: Supplementary Figure 5 — Cytokine-producing capability of in vitro-activated OT-I cells 3 days after adoptive transfer into HSV-OVA-infected hosts. Nucleofection of OT-I cells and viral infection were performed as in . (A, B) Representative flow cytometric plots of IFN-γ expression in viable OT-I cells (A) and TNF-α and IL-2 expression among IFN-γ+ OT-I cells (B) after 5 hr restimulation with 1 µM OVA257-264 (SIINFEKL) peptide in the presence of brefeldin A. Flow cytometric plots show concatenated data from one of two experiments with n = 5. (C, D) Summary of the frequency of IFN-γ+ (C) and IFN-γ+ TNF-α+ (C, F) cells among viable OT-I cells. Graphs show pooled data from two independent experiments with n = 9 or 15 for nucleofected cells or non-nucleofected control, respectively. Congenic marker assignment was swapped in each experiment. *p < 0.05, **p < 0.01, ***p < 0.001 as compared to NTC by ordinary one-way ANOVA test with Dunnett’s multiple comparison. [file Image_5.tif]

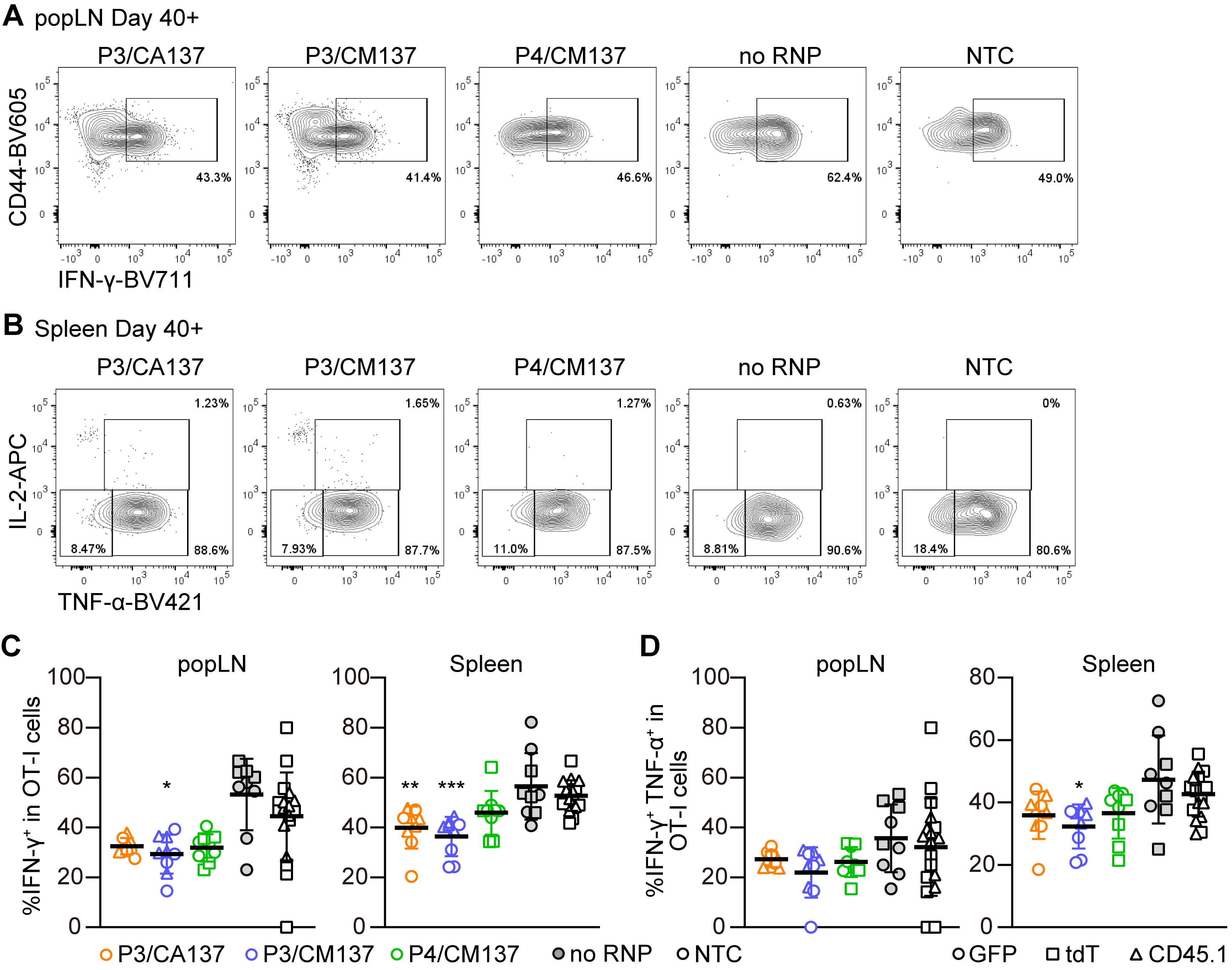

Supplement: Supplementary Figure 6 — Cytokine-producing capability of in vitro-activated OT-I cells >40 days after adoptive transfer into HSV-OVA-infected hosts. Nucleofection of OT-I cells and viral infection were performed as in Figure 2. (A, B) Representative flow cytometric plots of IFN-γ expression in viable OT-I cells (A) and TNF-α and IL-2 expression among IFN-γ+ OT-I cells (B) after 5 hr restimulation with 1 µM OVA257-264 (SIINFEKL) peptide in the presence of brefeldin A. Flow cytometric plots show concatenated data from one of two experiments with n = 5. (C, D) Summary of the frequency of IFN-γ+ (C) and IFN-γ+ TNF-α+ (C, F) cells among viable OT-I cells. Graphs show pooled data from two independent experiments with n = 9 or 15 for nucleofected cells or non-nucleofected control, respectively. Congenic marker assignment was swapped in each experiment. *p < 0.05, **p < 0.01, ***p < 0.001 as compared to NTC by ordinary one-way ANOVA test with Dunnett’s multiple comparison. [file Image_6.tif]

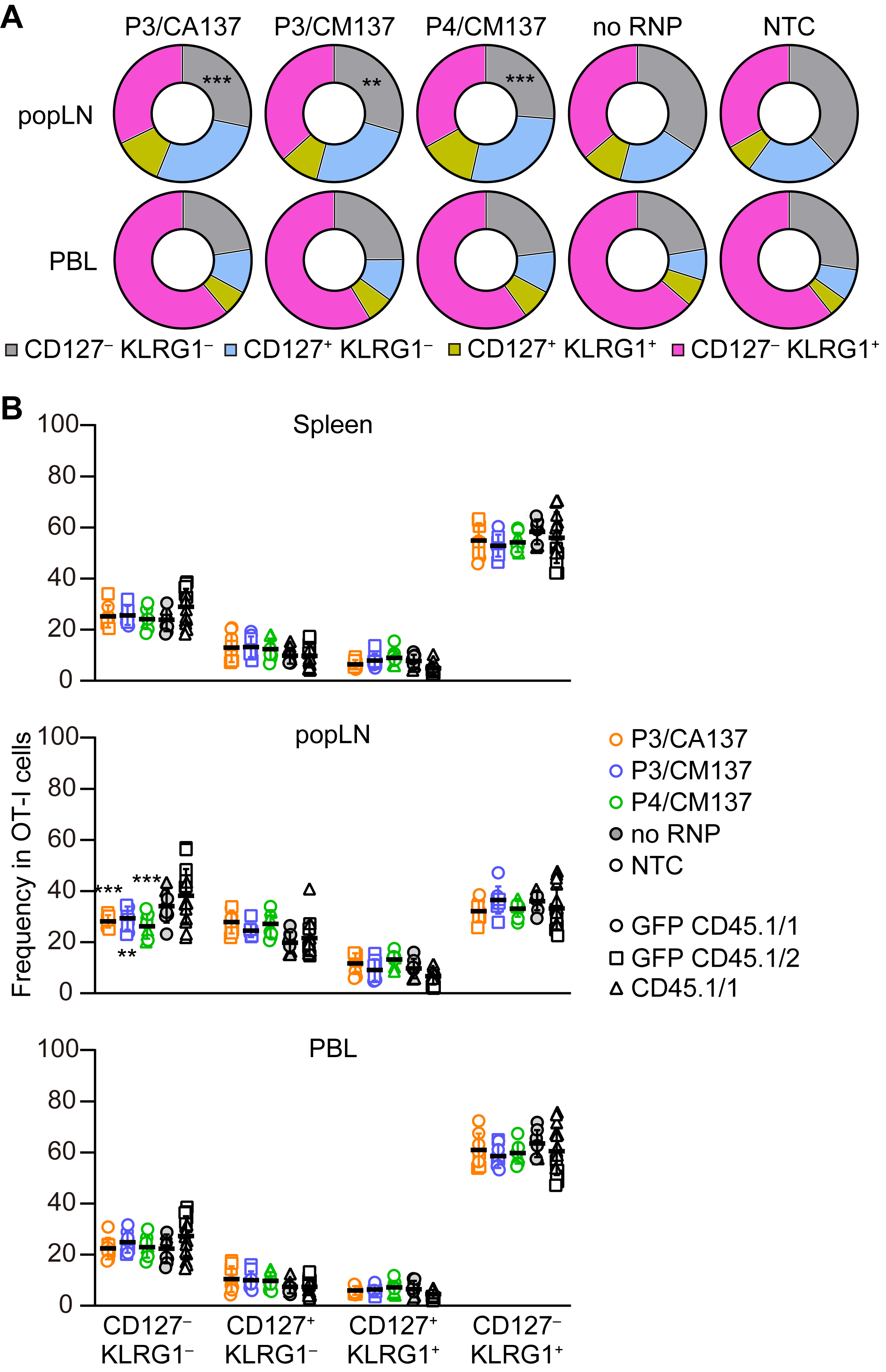

Supplement: Supplementary Figure 7 — CD127 and KLRG1 expression on OT-I cells in popLN and PBL after LCMV-OVA challenge. (A) Pie charts showing mean frequencies of four subsets identified by the expression pattern of CD127 and KLRG1 (as shown in ) in popLN and PBL. (B) Scatter plots of the data shown in (A) and . **p < 0.01, ***p < 0.001, ****p < 0.0001 by ordinary two-way ANOVA with Dunnett’s multiple comparison. [file Image_7.tif]

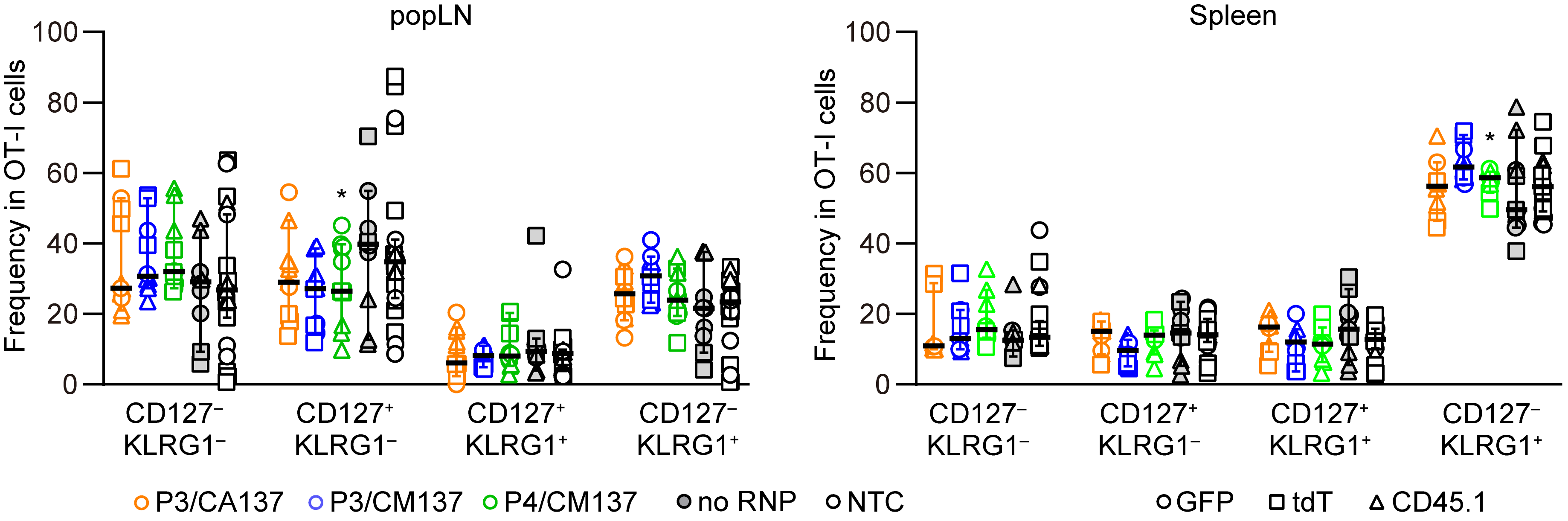

Supplement: Supplementary Figure 8 — Scatter plots of the data shown as pie charts in . Frequency of four subsets defined by the expression of CD127 and KLRG1 on day 7. *p < 0.05, **p < 0.01 as compared to NTC by ordinary two-way ANOVA with Dunnett’s multiple comparison. [file Image_8.tif]
